# Supplementary material for: Introduced Herbivores Threaten the Conservation Genetics of Two Critically Endangered Single-Island Endemics, Crambe sventenii and Pleudia herbanica
Source: Plants (Basel). 2024 Sep 13;13(18):2573. doi: 10.3390/plants13182573 (PMC11435307; doi:10.3390/plants13182573)
Supplement: Supplementary file 1 [file plants-13-02573-s001.zip › plants-3174316-supplementary.pdf]

Table S1. ***Crambe sventenii*** **and** ***Pleudia herbanica*** **populations sampled in Fuerteventura. x and y: Coordinates**

Species	Population	x	y	Population Code	Total number of individuals (2013)	Number of samples analysed
Crambe sventenii	Fuerteventura Botanical Garden			C-JBOT	-	20
	Montaña Cardón	28R 582329	3126678	C-CAR	17	18
	Risco colinos	28R 592082	3123322	C-COL	15	16
	Montañas de Vigán-El Roque	28R 601795	3125825	C-VIG	128	14
	Morro del Peñón	28R 602593	3128403	C-PEÑ	112	15
	Cuchillo de los Olivos	28R 603336	3129778	C-OLI	66	15
	Riscos del Saladillo	28R 603935	3132600	C-SAL	131	15
	Total				469	93
Pleudia herbanica	Fuerteventura Botanical Garden			P-JBOT	-	15
	Montaña Cardón	28R 581839	3124258	P-CAR	45	9
	Resbaladero Grande	28R 580699	3122819	P-RES	529	80
	Morro de Sise	28R 581860	3121165	P-SIS	5	2
	El Caracol	28R 589567	3122395	P-COL	3	6
	Cuchillo de Valle Largo	28R 592104	3122520	P-VLAR	390	50
	Montañas de Vigán-El Roque	28R 602046	3125582	P-VIG	54	16
	Morro del Peñón	28R 603017	3128100	P-PEÑ	5	4
	Cuchillo de los Olivos	28R 603371	3129752	P-OLI	32	9
	Riscos del Saladillo	28R 603517	3132304	P-SAL	36	12
	Atalaya de Pozo Negro	28R 604652	3132134	P-POZ	94	30
	Total				1193	234

Table S2. Characteristics of the microsatellite loci developed for *Crambe sventenii* and *Pleudia herbanica*. The markers were grouped in 4 Multiplex reactions in *C. sventenii* (A.B.C.D) and 3 reactions in *P. herbanica* (A.B.C).

Species	Locus	Genbank ID	Multiplex	Fluorophore	Primers 5'->3'	Motif	Range (bp)
Crambe sventenii	CSdi2	LS480642	A	D-PET	F: GCTAGGAAGCGAACGACGAT<br>R: AGACGAAACACATCGACCCA	(AG)20	119-137
	CSdi3	LS480643	D	B-VIC	F: GCATAGCCCTCTTCATCCCT<br>R: GCAGGGTGGTTATAGGCACA	(AG)19	167-213
	CSdi5	LS480644	B	C-NED	F: CCATTAGCGCAGAACCATGT<br>R: GACCAATGGTCCTGCCTTTG	(AG)12	132-193
	CSdi9	LS480645	C	D-PET	F: CTGAAGGCAGTGAGGGACG<br>R: TGCCCTATGATTTCGGCCAA	(AG)11	121-131
	CSdi15	LS480647	D	D-PET	F: GAGGCGCCATTAACGGAGAT<br>R: CCGGATATCGCCTGCTTTCA	(AG)9	238-244
	CStri1	LS480648	C	D-PET	F: TGATGAATGGAGCGGACACA<br>R: AAACCTGCGATCTGGAGACG	(AAG)10	182-191
	CStri2	LS480649	B	D-PET	F: CAGAAGGCTCTGGAGGTTGT<br>R: GAATTGGATGAACCGCTGCC	(ATC)9	165-189
	CStri3	LS480650	D	D-PET	F: GGGTTCCGTTTCGTTTCGTT<br>R: GGATCGTCGTTCGTTTGGGA	(AAG)9	149-158
	CStri5	LS480651	D	A-FAM	F: GGAAGAGGCCCTCGACAAAT<br>R: CGAGAGCTCAGACCTTCACA	(AAG)9	135-141
	CStri8	LS480652	B	C-NED	F: AGGATGAACATGTAGGGACGG<br>R: TCATCATCCACTTGCCAGCA	(AAG)9	237-243
	CStri9	LS480653	D	B-VIC	F: ACCTGGCTTGAGGTTGTTGA<br>R: TTCCGCTCCAAGTCCAAGTC	(AGG)8	116-122
	CStri13	LS480654	A	C-NED	F: GAGTCAACATACGAGCTGGGT<br>R: TTTGCGACGGCGAAGAGATC	(AAG)8	113-131
	CStri16	LS480655	C	B-VIC	F: GCGTGAAACTGAGTCCAGGA	(AGG)7	149-164

					R: ATCCATCGCTCTCCCTTTCG		
	CSpen1	LS480656	C	C-NED	F: CTGACTGCACCAAACCCAGA	(AGATC)6	125-135
					R: TCATCACCGTGAAGCTCTGC		
	CShex1	LS480658	A	A-FAM	F: TGGACGAGGAAGAGGAAGAGA	(AAGAGG)10	99-140
					R: ATCCTTCCTCTACTCCCGCC		
	CShex2	LS480657	B	A-FAM	F: GCTCCAGCTCCAACTCTAGC	(AAGCTC)8	138-169
					R: CGTACATGCTGTGCTTGAGC		
	CShex3	LS480659	A	A-FAM	F: TCCGCTCGCTTGTTTCAGAT	(ACAGAG)6	294-320
					R: CACAATCTGGCCTGCCATTG		
	CSHex4	LS480660	B	B-VIC	F: CCCTCTCCTATTCGGCTCCT	(AAACCC)6	87-105
					R: CGTGTTCATCGATGGGACGA		
	CShex5	LS480661	A	B-VIC	F: GTTTGCCTTATCCGTTCCGC	(AAGATG)6	110-153
					R: ACCATCTGCGCCATTGTTCT		
	CShex7	LS480662	D	C-NED	F: ATCTCTAGCTCAGCGGAAGG	(AAGAGG)5	101-113
					R: GCCAAGAGACAGCCCAAGAT		
Pleudia herbanica	SHEdi3	LS999439	C	B-VIC	F: CCAAGTCCAACGCTAAAGCC	(AG)18	120-128
					R: TGAGGGAGAAGAGAACGGGA		
	SHEdi4	LS999440	B	C-NED	F: AGGACCAGCTGGATGATTCC	(AG)17	170-186
					R: CCCGTGGCCGTATTTGTTGA		
	SHEdi5	LS999441	A	B-VIC	F: GCAGCATGCATGACATTCCC	(AG)16	99-109
					R: TTTGCTGGTTACATGTGAGCA		
	SHEdi6	LS999442	B	D-PET	F: CTCCTCTGGTGATGTCTGCG	(AG)16	246-250
					TGAGGCGATATCCCTACCATTC		
	SHEdi15	LS999444	A	D-PET	F: GGACATGTGTAAACGGTGTGC	(AT)8	139-141
					R: CCAGCACATGATGTCAAGCA		
	SHEtri1	LS999445	A	C-NED	F: TGCGTGCTCAAAGAAATGAACA	(AAT)15	155-179
					R: CGTATGACAGTTTGCGACCG		
	SHEtri2	LS999446	C	C-NED	F: TGGAGCAAGTTGGGAAGACA	(AAG)12	194-215
					R: TGGGACGGAGGGAGTATCAT		

SHEtri6	LS999448	A	C-NED	F: AAGGCTGCTTCTTGTGGTCG<br>R: AGCTGCTCCAGAACATCTCG	(AAG)8	230-236
SHEtri7	LS999449	B	A-FAM	F: TGCAGGCGAGCAAATGTAGT<br>R: CAGACTGAAGCGGGCATATCT	(AGC)8	126-132
SHEtri8	LS999450	B	C-NED	F: GGCTCGAAGCGATTAGGGTT<br>R: CCACAAAGCCCAGATTTCGT	(AAG)8	115-118
SHEtri9	LS999451	C	A-FAM	F: TCACAGCGCTCGTCATGTT<br>R: AGAAGGGTACAATCGAGCGAC	(AGC)8	98-104
SHEtri11	LS999452	A	A-FAM	F: GGTGATCGGATCCATAGGCG<br>R: ACCGACAACAACTGCGTACT	(ACC)7	117-126
SHEtri19	LS999453	A	A-FAM	F: CCACCATCCAACGGCTCATA<br>R: ACCTTCTCCCGATGACGAGA	(CCG)6	176-179
SHEtri20	LS999454	C	C-NED	F: GCCTCTCCCAATTGTCCACA<br>R: GCGGCGGAAGTTAAGGAGAT	(AAG)6	103-106
SHEtet1	LS999455	C	A-FAM	F: CCACCAGCTGCAAAGTCGAA<br>R: CATACGTTGACTCACCGGCT	(AACC)6	158-166

Table S3. Allele frequencies of *Crambe sventenii* populations. Private alleles are grey coloured. Population codes are indicated in Table 1.

Locus	Allele	C-JBOT	C-SAL	C-OLI	C-PEÑ	C-VIG	C-COL	C-CAR
**CShex1**	**99**	0.350	0.200	0.700	0.100	0.964	0.688	
	**117**	0.650	0.367					1.000
	**122**		0.267					
	**128**						0.313	
	**134**		0.167	0.300	0.700			
	**140**				0.200	0.036		
**CShex3**	**294**	0.389	0.133	0.967	0.333	0.143		0.556
	**300**		0.100					
	**305**	0.222	0.033					0.444
	**309**		0.600	0.033	0.667	0.25		
	**311**		0.133					
	**318**	0.389				0.607	0.938	
	**320**						0.063	
**CShex5**	**110**	0.550	0.700	0.067	0.933	0.321		0.972
	**128**		0.133				0.844	
	**134**	0.450		0.933	0.067	0.321		
	**141**							0.028
	**147**		0.133			0.357	0.156	
	**153**		0.033					
**CStri13**	**113**		0.600	0.067	0.100	1.000	1.000	
	**122**		0.067	0.800	0.867			
	**125**	0.650		0.133	0.033			0.722
	**128**	0.350	0.133					0.278
	**131**		0.200					
**CSdi2**	**119**	0.600	0.933	0.467	0.533	0.929		

	**121**			0.533	0.467	0.071	0.156	
	**123**						0.844	
	**127**		0.067					
	**137**	0.400						1.000
**CShex2**	**138**		0.333	0.100				
	**144**					0.214		
	**151**	0.550	0.200		0.267	0.321		
	**157**	0.450	0.467	0.900	0.600		1.000	1.000
	**163**				0.133	0.429		
	**169**					0.036		
**CSHex4**	**87**	0.800	0.433	1.000	0.800	1.000	1.000	
	**93**		0.567					
	**105**	0.200			0.200			1.000
**CSdi5**	**132**			0.233	0.100	0.321		
	**141**	0.850	0.033			0.036	1.000	1.000
	**154**		0.333					
	**158**		0.500		0.267			
	**164**			0.700	0.600	0.071		
	**166**					0.357		
	**173**	0.050			0.033	0.214		
	**175**	0.100	0.133					
	**193**			0.067				
**CStri8**	**237**	0.050						0.139
	**240**	0.900	0.667	0.667	0.767	0.679	1.000	0.861
	**243**	0.050	0.333	0.333	0.233	0.321		
**CStri2**	**165**	0.500			0.433			1.000
	**180**	0.200	0.867	1.000	0.567	1.000		
	**183**		0.133				1.000	

	**189**	0.300						
**CStri16**	**149**			0.833	0.233	0.286	0.438	
	**158**	0.600	0.167	0.033	0.067			0.250
	**164**	0.400	0.833	0.133	0.700	0.714	0.563	0.750
**CSpen1**	**125**		0.600	0.267	0.167	1.000	1.000	
	**130**	0.550	0.400	0.733	0.833			
	**135**	0.450						1.000
**CSdi9**	**121**		1.000	0.367	0.333	0.107		
	**123**	0.600					1.000	1.000
	**125**					0.071		
	**129**			0.033	0.600	0.821		
	**131**	0.400		0.600	0.067			
**CStri1**	**182**	0.050	0.833		0.267	0.821	1.000	
	**185**		0.167	0.433				
	**191**	0.950		0.567	0.733	0.179		1.000
**CStri5**	**135**	0.050	0.300		0.133	0.107	0.938	0.083
	**138**	0.350	0.567	0.167	0.833	0.214		
	**141**	0.600	0.133	0.833	0.033	0.679	0.063	0.917
**CStri9**	**116**	0.050	0.500	0.200	0.233	0.143		0.306
	**119**	0.250	0.267			0.250	0.031	0.694
	**122**	0.700	0.233	0.800	0.767	0.607	0.969	
**CSdi3**	**167**	0.650						1.000
	**195**				0.100	0.750	0.250	
	**197**		0.033					
	**199**			0.500	0.667	0.250	0.031	
	**201**						0.719	
	**203**	0.350	0.067					

	**207**		0.133					
	**211**		0.767	0.067	0.233			
	**213**			0.433				
**CShex7**	**101**	0.450						0.889
	**107**	0.500	0.300	0.633	0.567	0.821	1.000	0.111
	**113**	0.050	0.700	0.367	0.433	0.179		
**CStri3**	**149**	0.600	0.500		0.600	1.000	0.844	
	**152**	0.400	0.067					1.000
	**155**		0.233	1.000	0.400		0.156	
	**158**		0.200					
**CSdi15**	**238**	0.350	0.033					1.000
	**240**				0.233			
	**242**	0.150	0.967	1.000	0.767	0.250		
	**244**	0.500				0.750	1.000	

Table S4. Pairwise F_{ST} statistics for all the population comparisons in *Crambe sventenii*. Population codes are indicated in Table 1. All results are significant (p < 0.001)

	C-JBOT	C-SAL	C-OLI	C-PEÑ	C-VIG	C-COL
**C-SAL**	0.393	-				
**C-OLI**	0.445	0.448	-			
**C-PEÑ**	0.346	0.279	0.325	-		
**C-VIG**	0.424	0.37	0.509	0.406	-	
**C-COL**	0.586	0.601	0.708	0.63	0.568	-
**C-CAR**	0.438	0.668	0.749	0.664	0.753	0.841

Table S5. Allele frequencies of *Pleudia herbanica* populations and regions. Exclusive alleles among populations are grey coloured. Population codes are indicated in Table 1.

			WEST			SOUTH		EAST				
**Locus**	**Alleles**	**P-JBOT**	**P-CAR**	**P-RES**	**P-SIS**	**P-CCOL**	**P-VLAR**	**P-VIG**	**P-PEÑ**	**P-OLI**	**P-SAL**	**P-POZ**
**SHEdi3**	**120**	0.167	1.000	0.956	1.000	0.167	0.020					0.569
	**122**	0.567		0.044		0.833	0.800	1.000	1.000	1.000	1.000	0.431
	**126**	0.267					0.160					
	**128**						0.020					
**SHEdi4**	**170**	0.367						0.781	0.500	0.429	0.667	0.645
	**172**	0.367	0.778		1.000	0.750	0.670		0.500	0.286	0.292	0.258
	**182**		0.222	0.994		0.250	0.150					
	**184**						0.050	0.219		0.286	0.042	0.097
	**186**	0.267		0.006			0.130					

**SHEdi5**	**99**								1.000	0.111		
	**105**							0.375				
	**107**	0.400					0.620	0.375		0.500	0.958	0.29
	**109**	0.600	1.000	1.000	1.000	1.000	0.380	0.250		0.389	0.042	0.71
**SHEdi6**	**246**	0.400	1.000	0.944	0.500	0.750	0.271	0.750	1.000	0.714		
	**248**	0.600		0.044	0.500	0.250	0.625	0.219		0.286	1.000	0.967
	**250**			0.013			0.104	0.031				0.033
**SHEdi15**	**139**	0.333	1.000	0.931	1.000	1.000	0.790					
	**141**	0.667		0.069			0.210	1.000	1.000	1.000	1.000	1.000
**SHEtri1**	**155**			0.019								
	**161**						0.030			0.056		
	**164**	0.533		0.081				0.750		0.667		0.226
	**167**	0.067	1.000	0.900	0.500	0.583	0.320					
	**170**	0.367				0.417	0.510					
	**173**	0.033					0.100					0.371
	**176**						0.040		0.875	0.278	1.000	0.403
	**179**				0.500			0.250	0.125			
**SHEtri2**	**194**	0.200				0.417	0.350	0.250		0.056	0.292	0.724
	**200**		0.056	0.063		0.500					0.042	
	**206**	0.033		0.063								
	**209**	0.633	0.944	0.875	1.000		0.430	0.750	1.000	0.944	0.667	0.276
	**212**	0.133				0.083	0.200					
	**215**						0.020					

**SHEtri6**	**230**			0.340		0.417	0.440	0.250				
	**236**	1.000	1.000	0.660	1.000	0.583	0.560	0.750	1.000	1.000	1.000	1.000
**SHEtri7**	**126**	0.100					0.130			0.313		0.145
	**132**	0.900	1.000	1.000	1.000	1.000	0.870	1.000	1.000	0.688	1.000	0.855
**SHEtri8**	**115**	0.933	0.500	0.288	1.000	1.000	0.850	1.000	1.000	1.000	1.000	1.000
	**118**	0.067	0.500	0.713			0.150					
**SHEtri9**	**98**			0.131		0.500	0.250					
	**104**	1.000	1.000	0.869	1.000	0.500	0.750	1.000	1.000	1.000	1.000	1.000
**SHEtri11**	**117**	0.300						0.500		0.563		0.290
	**126**	0.700	1.000	1.000	1.000	1.000	1.000	0.500	1.000	0.438	1.000	0.710
**SHEtri19**	**176**	0.800	0.333	0.050		0.417	0.370	1.000	1.000	1.000	0.958	0.968
	**179**	0.200	0.667	0.950	1.000	0.583	0.630				0.042	0.032
**SHEtri20**	**103**	0.033			0.750		0.050		0.375	0.111		
	**106**	0.967	1.000	1.000	0.250	1.000	0.950	1.000	0.625	0.889	1.000	1.000
**SHEtet1**	**158**	0.767	0.500	1.000	1.000	0.833	0.630	1.000	1.000	1.000	1.000	1.000
	**166**	0.233	0.500			0.167	0.370					

Table S6. Pairwise F_{ST} statistics between the regions estimated in *Pleudia herbanica*. All results are significant ($p < 0.001$).

	WEST	SOUTH
**SOUTH**	0,436	
**EAST**	0,598	0,297

Figure S1. Output results from STRUCTURE HARVESTER for *Crambe sventenii* and *Pleudia herbanica*. A.1) The mean of log-likelihood values for each value of *K* (1-7) in *C. sventenii*; A.2) Ad hoc statistic based on the rate of change in the log probability of data between successive K values (ΔK) in *C. sventenii*, following Evanno et al. (2005) [38]. B.1) The mean of log-likelihood values for each value of *K* (1-10) in *P. herbanica*; B.2) Ad hoc statistic based on the rate of change in the log probability of data between successive K values (ΔK) in *P. herbanica*.

A.1)

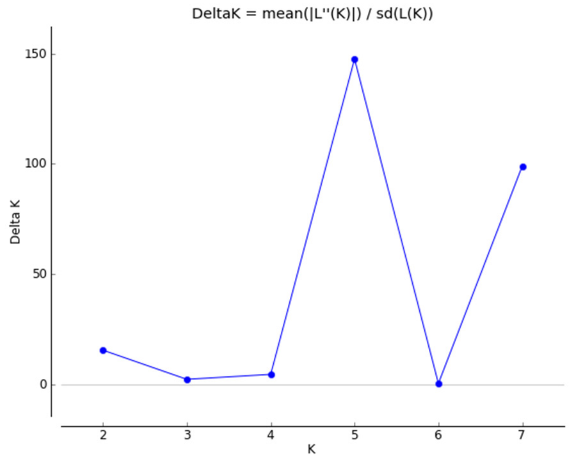

A.2)

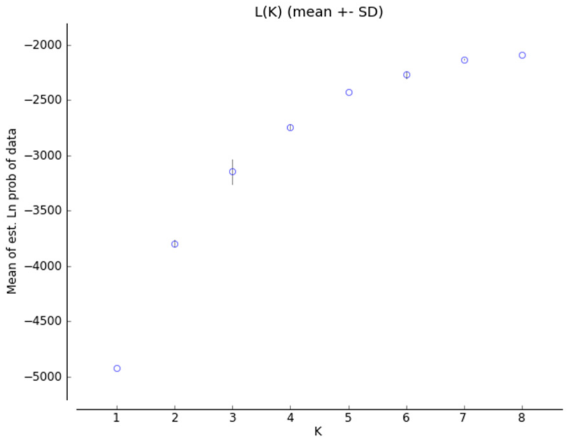

B.1)

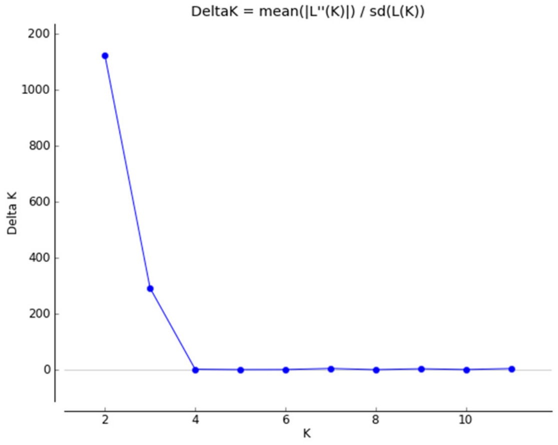

B.2)

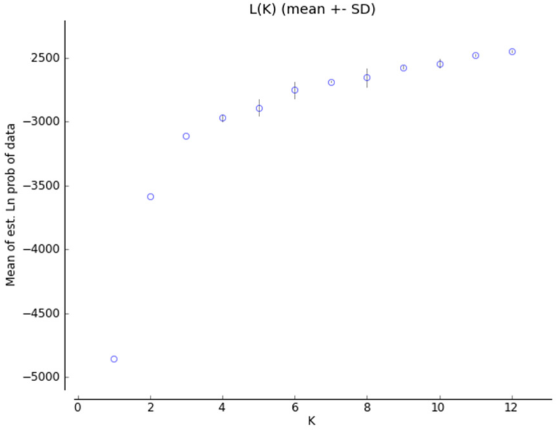

Figure S2. Relation of the geographic distance divided by ten spatial classes to the F_{ST}/1-F_{ST} values, in order to test for Isolation by Distance pattern in *Crambe sventenii* (A) and *Pleudia herbanica* (B)

(A)

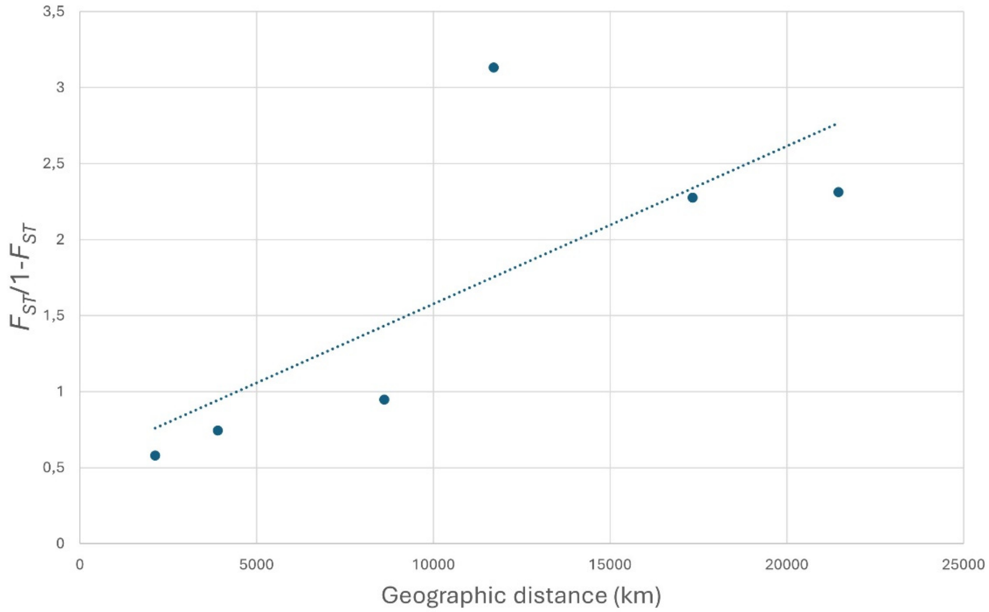

(B)

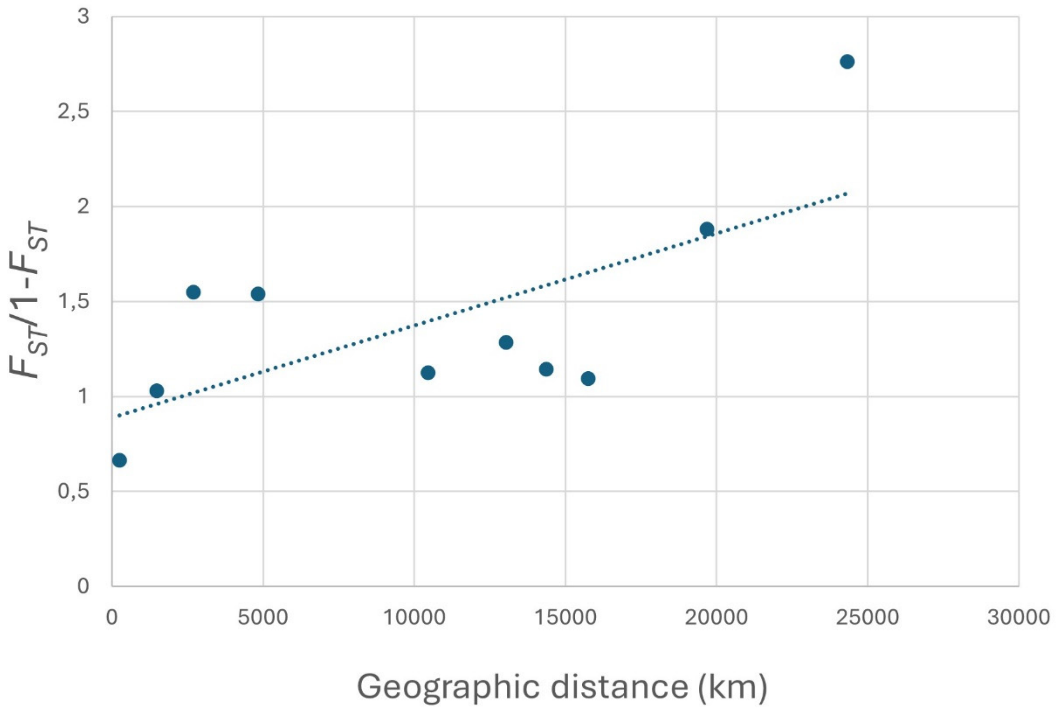

3
2,5
2
1,5
1
0,5
0
$F_{ST}/1-F_{ST}$
0
5000
10000
15000
20000
25000
30000
Geographic distance (km)

Figure S3. Principal Component Analysis (PCA) of the 35 topo-climatic variables tested for the *Crambe sventenii* (A) and *Pleudia herbanica* (B) niche modelling.

(A)

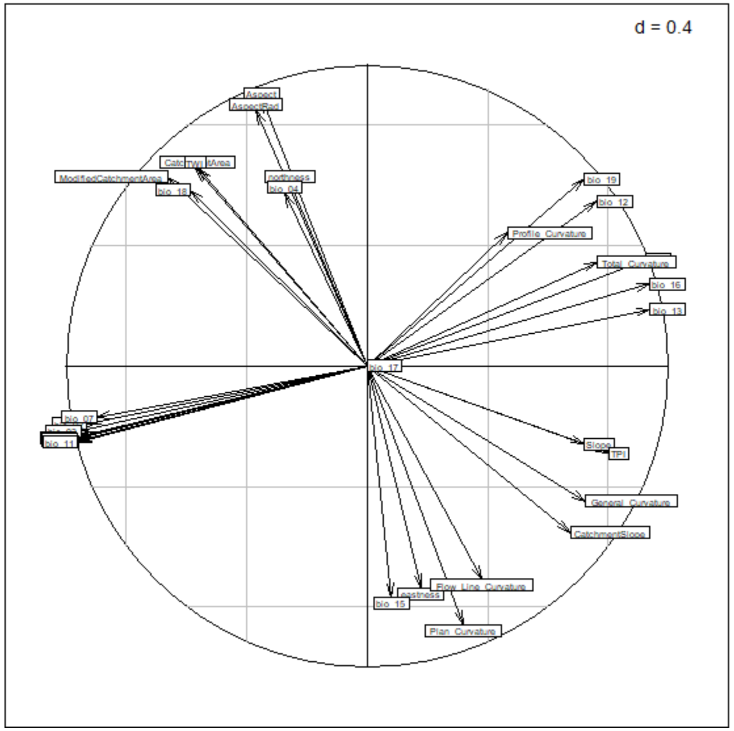

(B)

(B)

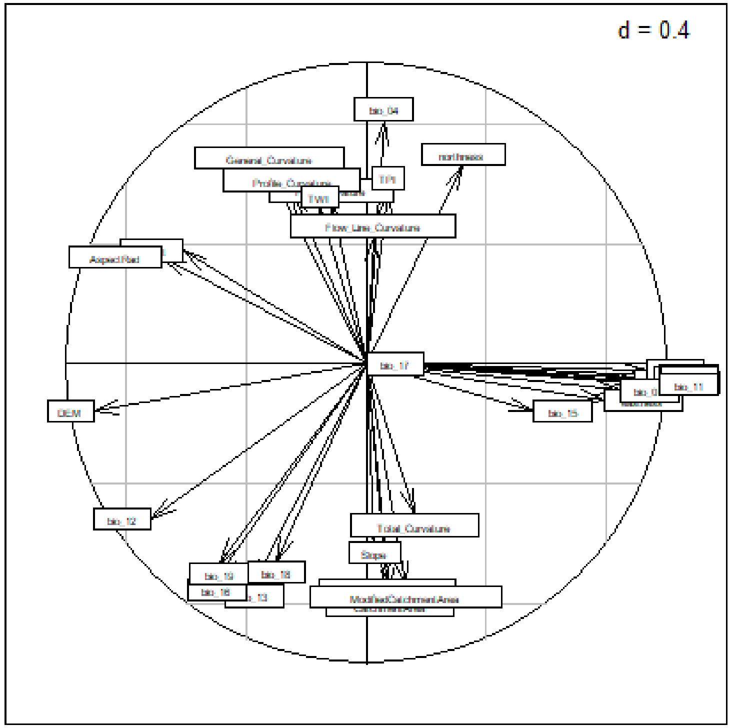

Figure S4. Dendrograms to detect multicollinearity among the topo-climatic variables for the *Crambe sventenii* (A) and *Pleudia herbanica* (B) niche modelling.

(A)

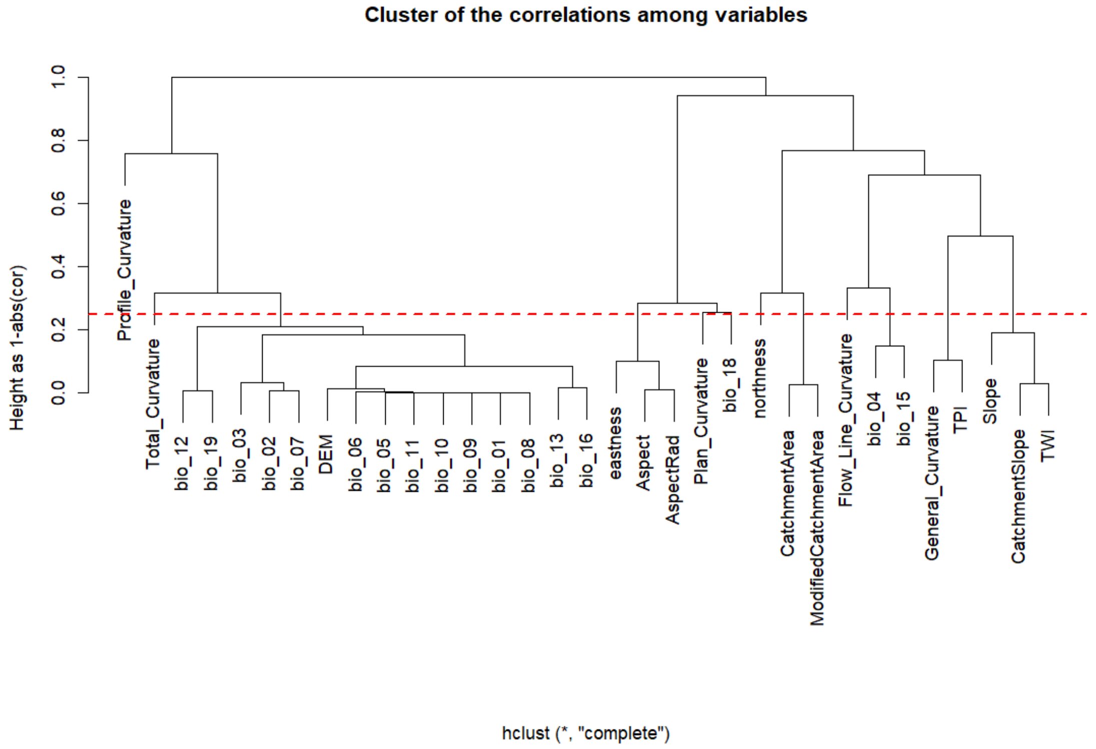

(B)

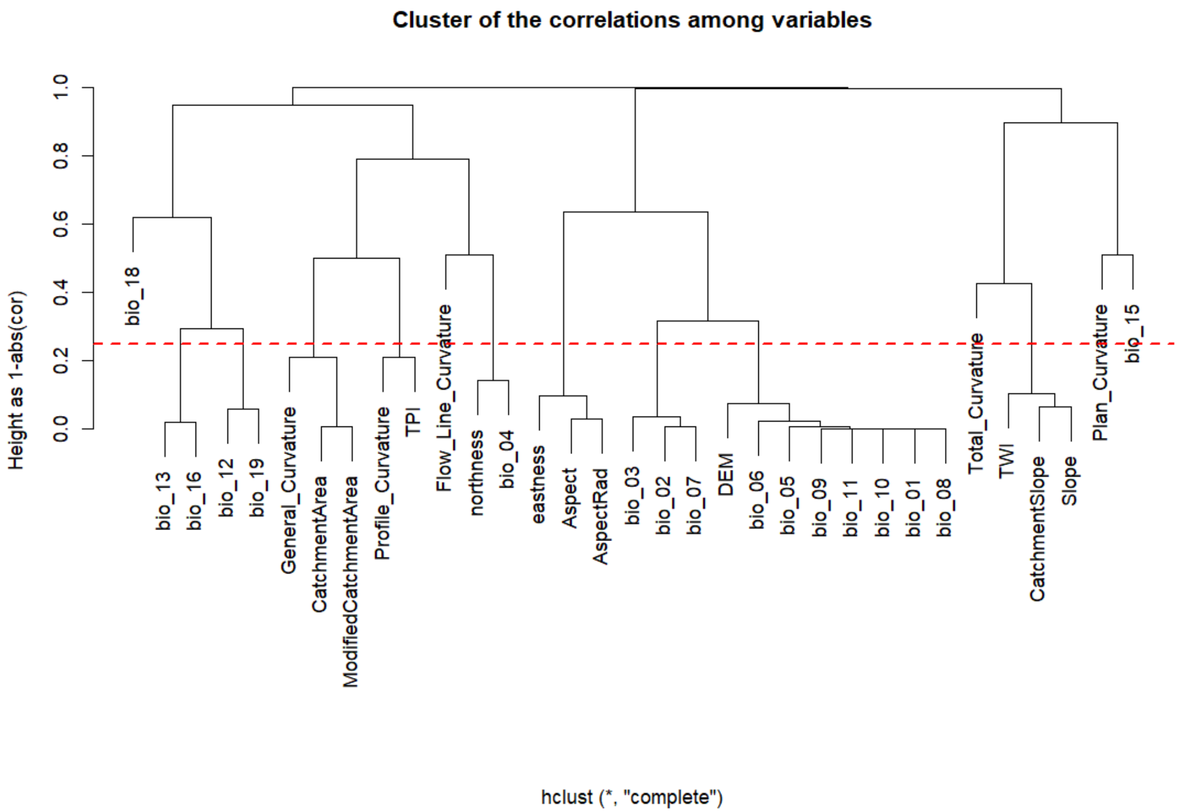
Cluster of the correlations among variables
Height as 1-abs(cor)
1.0
0.8
0.6
0.4
0.2
0.0
bio_18
bio_13
bio_16
bio_12
bio_19
General_Curvature
CatchmentArea
ModifiedCatchmentArea
Profile_Curvature
TPI
Flow_Line_Curvature
northness
bio_04
eastness
Aspect
AspectRad
bio_03
bio_02
bio_07
DEM
bio_06
bio_05
bio_09
bio_11
bio_10
bio_01
bio_08
Total_Curvature
TWI
CatchmentSlope
Slope
Plan_Curvature
bio_15
hclust (*, "complete")
